# Immunomodulatory Activity on Human Macrophages by Cell-Free Supernatants to Explore the Probiotic and Postbiotic Potential of *Lactiplantibacillus plantarum* Strains of Plant Origin

**DOI:** 10.1007/s12602-023-10084-4

**Published:** 2023-05-18

**Authors:** Maria Teresa Rocchetti, Pasquale Russo, Nicola De Simone, Vittorio Capozzi, Giuseppe Spano, Daniela Fiocco

**Affiliations:** 1 Department of Clinical and Experimental Medicine, University of Foggia, Foggia, Italy; 2 Department of Food, Environmental and Nutritional Sciences, University of Milan, Milan, Italy; 3 Department of Agriculture Food Natural Science Engineering (DAFNE), University of Foggia, Foggia, Italy; 4 Institute of Sciences of Food Production, National Research Council (CNR) of Italy, C/O CS-DAT, Foggia, Italy

**Keywords:** Cytokines, Lactobacilli, Caco-2, Adhesion, Immune response, Anti-inflammatory, Plant-associated

## Abstract

Upon dietary administration, probiotic microorganisms can reach as live cells the human gut, where they interact with the microbiota and host cells, thereby exerting a beneficial impact on host functions, mainly through immune-modulatory activities. Recently, attention has been drawn by postbiotics, i.e. non-viable probiotic microbes, including their metabolic products, which possess biological activities that benefit the host. *Lactiplantibacillus plantarum* is a bacterial species that comprises recognised probiotic strains. In this study, we investigated in vitro the probiotic (and postbiotic) potential of seven *L. plantarum* strains, including five newly isolated from plant-related niches. The strains were shown to possess some basic probiotic attributes, including tolerance to the gastrointestinal environment, adhesion to the intestinal epithelium and safety. Besides, their cell-free culture supernatants modulated cytokine patterns in human macrophages in vitro, promoting TNF-α gene transcription and secretion, while attenuating the transcriptional activation and secretion of both TNF-α and IL-8 in response to a pro-inflammatory signal, and enhancing the production of IL-10. Some strains induced a high IL-10/IL-12 ratio that may correlate to an anti-inflammatory capacity in vivo. Overall, the investigated strains are good probiotic candidates, whose postbiotic fraction exhibits immunomodulatory properties that need further in vivo studies. The main novelty of this work consists in the polyphasic characterisation of candidate beneficial *L. plantarum* strains obtained from relatively atypical plant-associated niches, by an approach that explores both probiotic and postbiotic potentials, in particular studying the effect of microbial culture-conditioned media on cytokine pattern, analysed at both transcriptional and secretion level in human macrophages.

**Supplementary Information:**

The online version contains supplementary material available at 10.1007/s12602-023-10084-4.

## Introduction

Probiotics are live microorganisms that are beneficial to the host [1]. The health benefits driven by probiotics are strain dependent and need to be ascertained by appropriate experimentations. Although specific probiotic claims need to be proved by large observational studies and/or properly controlled clinical trials [1], an initial characterisation of candidate probiotics can rely on more straightforward in vitro analyses [2, 3] which help to preliminarily evaluate some desirable properties, such as safety, antagonism against pathogens, survival to gastrointestinal transit, ability to colonise the intestine and immunomodulatory activity. The concept of probiotic can be accompanied by a possible evolution in terms of postbiotic potential, intended as a “health benefit on the host” addressable to a “preparation of inanimate microorganisms and/or their components” [4, 5]. Indeed, health-promoting features do not depend only on metabolically active, viable microbial cells, as they may also be ascribed to probiotic-derived molecules [6–8], including the mixture of metabolites secreted into the culture medium, often referred to as cell-free culture supernatants (CFS) [9, 10], or released together with cell structural components, upon cell lysis or inactivation [11, 12]. Interestingly, probiotic-derived molecules often share the same health potential as probiotics and, additionally, have some advantages over the limitations of the latter [13]. In this light, the study of the immunomodulatory properties of CFS provides insight into the probiotic behaviour, but also preliminary information concerning a postbiotic potential. Among others, probiotics and postbiotics have been shown to exert therapeutic effects against inflammation-related disorders, including inflammatory bowel disease [14–16]. Such therapeutic properties depend mainly, though not solely, on their immunomodulatory capacity [17, 18], i.e. their ability to interact with host immune-competent cells, thereby influencing their behaviour and development, including the production and release of immune mediators [12, 19–22].

Probiotic bacterial strains often belong to the family of *Lactobacillaceae* and to *Bifidobacterium* species [23]. *Lactobacillaceae* are part of the lactic acid bacteria (LAB), a vast and diverse group of non-sporulating Gram-positive, which inhabit soil, plants, food matrices and animal mucosal surfaces, including the human gut [24]. Recently, the “proximity” of LAB strains from food environments and intestinal niche has been highlighted, pointing to the food microbiota as a possible source of LAB for the intestinal tract microbiome [24]. Several LAB species are employed for food production and preservation [25], hold potential for biomedical purposes [26, 27] and enjoy safety attributes recognised by both the European Food Safety Authority (EFSA) and the Food and Drug Administration (FDA) [28]. Together with other members of the gut microbiota, LAB play a relevant role in the immune homeostasis of the host [29]. Indeed, their cell surface components (e.g. lipoteichoic acids, LTA), as well as secreted metabolites, can be recognised by pattern recognition receptors (PRRs) involved in innate immunity, thereby modulating inflammatory signaling and influencing the maturation and functions of immune cells [30, 31].

*Lactiplantibacillus plantarum* is a widespread and versatile LAB species, which is extensively used both in food technologies [32] and for probiotic applications [33, 34]. Compared to other LAB, *L. plantarum* has a large genome and can adapt to a variety of habitats, such as different types of fermented foods, vegetables, fruits, the gut of both vertebrates and invertebrates, and the mucosa of human uro-genital and upper respiratory tracts [35–37]*. L. plantarum* has been safely used for centuries in food productions; moreover, several strains have been found to exhibit relevant probiotic attributes, including tolerance to acid and bile, persistence in the human gastrointestinal tract [38], inhibition of pathogens [39] and the ability to prevent intestinal dysbiosis, while promoting gut barrier function [40, 41]. Indeed, animal studies and clinical trials have pointed to the health benefits deriving from *L. plantarum* administration, particularly in the context of gastrointestinal and inflammatory disorders [42].

So far, several LAB have been characterised in relation to their health-promoting characteristics; however, the search for novel probiotics remains of interest [43]. Usually, *L. plantarum* probiotic strains are either of human origin or derived from dairy-associated environments [33, 44]. In this work, we assessed the immunomodulatory properties of cell-free culture supernatants (CFS) from seven candidate probiotic strains of *L. plantarum* derived from plant-related matrices, including five new isolates from unusual fruit and plant-related niches and two strains previously selected for their antimicrobial and antiviral properties.

## Materials and Methods

### Reagents

Dulbecco’s modified Eagle’s medium (DMEM), Roswell Park Memorial Institute medium (RPMI), trypsin–EDTA were from Gibco (Carlsbad, CA, USA). Bile salts, 3-(4,5-dimethylthiazol-2-yl)-2,5-diphenyl tetrazolium bromide (MTT), dimethyl sulfoxide (DMSO), foetal bovine serum, L-glutamine, lipopolysaccharides (LPS) from *Escherichia coli* O127:B8, lysozyme, pancreatin, penicillin, pepsin, phorbol 12-myristate 13-acetate (PMA) and streptomycin were purchased from Sigma-Aldrich (St Louis, MO, USA); de Man-Rogosa-Sharpe (MRS) broth (Biolife Italiana, Milano, Italy); tryptone soya broth (TSB), and Brain Heart Infusion broth (BHI) were purchased from Oxoid (Basingstoke, UK).

### Isolation and Cultivation of Presumptive LAB and Selected Investigated Strains

LAB strains were isolated from spontaneous plant specimens (i.e. medlar, aloe, carob, mulberry and strawberry tree) and artisanal sourdoughs (Apulia, Italy). The isolation matrices (10 g) were aseptically resuspended in 90 mL of peptone water and homogenised using a stomacher. Serial dilutions of the homogenates were spread onto de MRS supplemented with CaCO_3_ (1.5 w/v) and incubated at 37 °C for 48 h in aerobic conditions. Presumptive LAB showing a clear zone around the colonies were isolated and cryoconserved in MRS containing 20% (v/v) of glycerol. The complete list of the isolated LAB strains is reported in Table S1. Among the isolated ones, the selected strains 10A, 11A, CB-56, CZ-97, CZ-103, and *L. plantarum* UFG121 [45] and NC8 [46] were grown in MRS at 37 °C for 18 h. CFS from *L. plantarum* stationary phase cultures were obtained by centrifugation at 10,000 × rpm for 1 min, and filtration on 0.22 μm filters. CFS were aliquoted and stored at − 30 °C until use.

### Microbial Strains for Antagonistic Assays

Three pathogenic bacterial strains, namely *Escherichia coli* O157:H7 UFG77, methicillin-resistant *Staphylococcus aureus* UFG141 and *Listeria monocytogenes* CECT 4031, were cultured in Tryptone Soya Broth (TSB) at 37 °C.

### Screening for Antimicrobial Activity

The antimicrobial activity of presumptive LAB isolates was determined against pathogenic bacteria using the overlay method, as previously reported [45]. Briefly, cultures at late exponential phase (corresponding to about 2 × 10^9^ colony-forming unit (CFU) mL^−1^, according to previously analysed growth curves) were spotted (5 µL, i.e. approximately 1 × 10^7^ CFU) on MRS agar plates and grown at 37 °C for 24 h. Then, plates were overlaid with 10 mL of TSB soft agar (0.75% w/v of agar) containing 10^6^ CFU mL^−1^ of the target bacterial pathogen. After 48-h incubation at 37 °C, the size of the inhibition zone around the spots was measured. Isolated LAB strains were classified as having no (-), low ( +), mild (+ +), or strong (+ + +) antimicrobial activity, if the inhibition zones were less than 1 mm, between 1 and 3 mm, between 3 and 5 mm, or greater than 5 mm, respectively [3].

### Molecular Identification of the Investigated Isolates

Genomic DNA was extracted using a microbial DNA isolation kit (Mobio Laboratories, Inc. Carlsbad, CA, USA) following manufacturer’s instructions. 16S rRNA gene sequences were amplified with 0.2 nM primer oligonucleotides BSF8 (5´-AGAGTTTGATCCTGGCTCAG-3´) and BSR1541 (5´-AAGGAGGTGATCCAGCCGCA-3´), 10 µM dNTPs and 2.5 U Taq polymerase (Qiagen, Hilden, Germany). The thermal cycling included denaturation at 94 °C for 4 min, followed by 30 cycles at 94 °C for 30 s, 55 °C for 30 s, 72 °C for 90 s, and a final extension at 72 °C for 5 min. Amplicons were checked by agarose gel electrophoresis, purified using the QIAquick PCR purification kit (Qiagen) and sequenced (Macrogen, Madrid, Spain). The species were identified by homology search using Basic Local Alignment Search Tool (BLAST, http://www.ncbi.nlm.nih.gov/BLAST). The 16S rRNA sequences of the investigated selected *L. plantarum* isolates were submitted to GenBank (https://submit.ncbi.nlm.nih.gov/) under the following accession numbers: ON584756, *L. plantarum* strain 10A; ON584769, *L. plantarum* strain 11A; ON585118, *L. plantarum* strain CB-56; ON585707, *L. plantarum* strain CZ-97; ON598622, *L. plantarum* strain CZ-103.

### In Vitro Survival in Simulated Oro-gastrointestinal Transit

Mid-exponential phase cultures of *L. plantarum* strains (OD_600nm_ = 0.8) were centrifuged (5000 g × 3 min) and resuspended into sterile saline solution (NaCl 8.6 g L^−1^) at a concentration of about 2 × 10^9^ CFU mL^−1^. The bacterial suspensions (t0) were subjected to a model mimicking the oro-gastrointestinal conditions, as reported in De Simone et al. [47]. Briefly, oral stress (t1) was simulated by adding 15 mg L^−1^ of lysozyme to a gastric electrolyte solution (6.2 g L^−1^ NaCl; 2.2 g L^−1^ KCl; 0.22 g L^−1^ CaCl_2_; 1.2 g L^−1^ NaHCO_3_) with pH 6.0, and incubating for 3 min at 37 °C. Then, 3 g L^−1^ pepsin was added, and the solution was acidified to pH 3.0 (t2) and then to 2.0 (t3), being each step incubated for 30 min at 37 °C. Then, the intestinal compartment was simulated by neutralising the solution to reach pH 6.5 and by adding porcine bile salts (3 g L^−1^) and pancreatin (1 g L^−1^), and incubating for 1 h at 37 °C (t4). Finally, samples were diluted (1:1, v/v) with an intestinal electrolyte solution (5 g L^−1^ NaCl; 0.6 g L^−1^ KCl; 0.25 g L^−1^ CaCl_2_) to mimic the large intestine and incubated for 1 h at 37 °C (t5). Dilutions from the different steps (t0t5) were plated on MRS agar to determine viable cells. The assays were performed in triplicate.

### Caco-2 Adhesion Assay

Caco-2 cells (HTB − ֿֿ37 – ATCC) were a kind gift from a lab colleague and were used between passage 20 and 30. Supplemented DMEM containing 10% (*v*/*v*) heat-inactivated fetal bovine serum, 2 mM L-glutamine, 50 U mL^−1^ penicillin and 50 μg mL^−1^ streptomycin was used to grow Caco-2 cells, at 37 °C with 5% CO_2_, in tissue culture-treated plates. Cell culture splitting was performed when 70–80% confluence was reached. In order to obtain steady monolayers, Caco-2 cells were seeded in 96-well plates at a concentration of 10^5^ cells mL^−1^ and grown for 2 weeks, changing the medium every 2 days. Adhesion assays were performed as previously described [3]. Briefly, absolute DMEM was used to replace the growth medium 24 h prior to the adhesion assay. One hundred microliters of mid-exponential phase cultures (OD_600 nm_ = 0.6–0.8, corresponding to 5 × 10^8^ CFU mL^−1^) from each *L. plantarum* strain were centrifuged, resuspended in DMEM and incubated with Caco-2 cells (0.1 mL per well) for 1 h, at 37 °C, with 5% CO_2_ (ratio 1000:1, bacteria to Caco-2 cells). After PBS washing, Caco-2 cells and adherent bacteria were detached from wells by addition of trypsin and re-suspended in PBS. To determine the number of cell-attached bacteria, the cell suspension was serially diluted and plated onto MRS agar for CFU counting. To calculate the adhesion’s percentages, CFU deriving from washed wells, containing only cell-bound bacteria, were compared with those from control unwashed wells, i.e. containing both unbound and bound bacteria. At least three independent experiments, with triplicate determinations, were conducted.

### Biofilm Formation

The biofilm-forming ability was investigated as previously described [3]. Briefly, *L. plantarum* strains were cultivated in 96-well plates for 5 days at 30 °C. Then, wells were washed and stained with 0.05% (w/v) crystal violet which was dissolved in 96% ethanol. Absorbance was collected at 570 nm. Assays were performed in triplicate.

### Antibiotic Resistance

The antibiotic susceptibility of tested *L. plantarum* strains was measured by the agar overlay diffusion method using a quantitative method consisting of MIC (minimum inhibitory concentration) test strip (Liofilmchem MTSTM, Waltham, MA, US) containing a gradient (0.016–256 mg L^−1^) of each of the following antibiotics: chloramphenicol, erythromycin, vancomycin, gentamycin, tetracycline, ampicillin, clindamycin, streptomycin and kanamycin. Cell suspensions from each strain were prepared to achieve a density of OD_600nm_ = 0.6, corresponding to 5 × 10^8^ CFU mL^−1^, as indicated by the manufacturer’s instructions. MRS agar plates were overlaid with 200 μL of suspension from each strain, and then the strip was placed on the plate surface and incubated at 30 °C for 16–24 h. MIC were determined by observing the inhibition ellipse intersecting the strip, as indicated by the manufacturer’s instructions. No inhibition ellipse indicated no growth of the tested *L. plantarum* strain to the highest value of indicated antibiotic (256 μg mL^−1^). Resistance and susceptibility of the tested strain were determined according to EFSA guidelines [48].

### Immuno-modulation of Macrophages

CFS of the different *L. plantarum* strains were tested for their ability to modulate the immune response of THP-1-derived macrophages. The suitable CFS concentration to be used for the immuno-modulation test was determined by a cytotoxicity test.

#### Cytotoxicity MTT-Based Assay

Human monocytoid leukaemia-derived cells (THP-1)*,* from Sigma-Aldrich, used at passage 10–20, were grown in RPMI supplemented with 10% (v/v) heat-inactivated fetal bovine serum, L-glutamine (2 mM), penicillin (50 U mL^−1^) and streptomycin (50 μg mL^−1^), at 37 °C in a humidified atmosphere (5% CO_2_). THP-1 cells were propagated keeping a density of 2.5 × 10^5^ cell mL^−1^. THP-1 were differentiated into macrophages by the addition of PMA (100 ng mL^−1^, for 48 h).

For MTT assay, macrophages were cultivated at a density 5 × 10^4^ cells per well, in 96-well cell culture plates. One hundred microliters of different CFS dilutions (50, 20, 15, 10 and 5%, (v/v)) in serum-free RPMI, for each strain, were added to each macrophage-containing well and incubated for 24 h at 37 °C and 5% CO_2_. After treatment, the medium was removed, wells were washed with PBS, and 100 μL of 0.5 mg mL^−1^ MTT in serum-free medium was added to each well, incubating for 4 h, at 37 °C, with 5% CO_2_. After removing supernatants, 100 μL of DMSO was added to dissolve and visualise the formazan crystals formed in living macrophages. To calculate the relative cell viability (%), the absorbance (595 nm) was read by a microplate reader (FilterMax F5, Molecular Devices, CA, USA). CFS-untreated macrophages were used as positive control, defining 100% viability. To calculate the relative cell viability (RCV), the following equation was used:

$$\mathrm{RCV}\;(\%)=\lbrack({\mathrm{OD}}_{595}\;\mathrm{sample}-{\mathrm{OD}}_{595}\mathrm{blank})/({\mathrm{OD}}_{595}\;\mathrm{control}-{\mathrm{OD}}_{595}\mathrm{blank})\rbrack\times100.$$

#### Cytokine Gene Expression

THP-1-derived macrophages were stimulated with 200 ng mL^−1^ LPS after pre-incubation with bacterial CFS or absolute RPMI (positive control). In detail, 5 × 10^5^ macrophages/well were seeded on a 24-well plate and incubated with 10% (v/v) CFS from each *L. plantarum* strain for 20 h (37 °C, 5% CO_2_). Soon after, LPS was added, and macrophages were further incubated for 3.5 h. Negative control was represented by CFS-untreated macrophages not incubated with LPS. TRIzol reagent (Invitrogen, Carlsbad, CA, USA) was used to extract macrophages total RNA, which was quantified and checked for integrity (NanoDrop™ V. 3.7.0, Thermo Scientific, Waltham, MA, USA), and subsequently, reverse-transcribed using QuantiTect^®^ Reverse Transcription kit (Qiagen, Valencia, CA, USA). Quantitative RT-PCR was performed for the transcriptional analysis of genes encoding tumour necrosis factor-alpha (TNF-α), interleukin 8 (IL-8), interleukin 10 (IL-10) and interleukin 12 (IL-12), by using QuantiFastSybr^®^ Green PCR kit (Qiagen) in a real-time instrument (ABI 7300, Applied Biosystem, Foster City, CA, USA), by applying the ΔΔCt method. β-Actin and glyceraldehyde-3-phosphate dehydrogenase (GAPDH) transcript levels were used as internal normaliser, as previously described [47, 49].

#### Cytokine Measurement

The concentrations of TNF-α, IL-8, IL-10 and IL-12, in the supernatants of the macrophages, treated with bacterial CFS, with or without LPS addition, were assayed by human ELISA kits according to the manufacturer’s protocol (Invitrogen, Life Technologies, Carlsbad, CA, USA). The optical densities (OD) at 450 nm were determined using a microplate reader (FilterMax F5, Molecular Devices, CA, USA).

### Statistical Analysis

Data are expressed as mean ± standard deviation (SD). Significant differences of variables among strains were assessed using one-way analysis of variance (ANOVA) followed by Fishers least significant difference (LSD) test, and Student’s *t* test, with *p* < 0.05 as the minimal level of significance. For all statistics, the Statview software package SAS (v. 5.0) was employed.

## Results

### Selection and Identification of Isolates for Probiotic Investigations

Fifteen presumptive LAB isolates were preliminarily screened by evaluating their antagonistic activity against representative food-borne pathogens [50]. All isolated strains, derived from different plant matrices, were tested by the overlay assay (supplementary Table S1). Based on previously proposed classifications [45], the isolates exhibited an overall moderate to strong antagonistic activity, with radii of inhibition zones ranging from 2.5 to 6.0 mm. *L. monocytogenes* CECT 4031 was quite a sensitive target, whereas the most resistant species was *E. coli* UFG77, whose growth was strongly inhibited only by strawberry tree fruits-derived strains, namely CZ97 and CZ103. Out of fifteen total isolates, five strains, i.e. 10A, 11A, CB56, CZ-97, CZ-103, identified as *L. plantarum* by rRNA 16S gene sequencing, exhibited a broader and higher antibacterial activity compared to the other (Table S1), and hence were selected for further studying their probiotic activity.

Together with new strains isolated from atypical plant-related niches of food interest, the probiotic characteristics were also studied in two other *L. plantarum* strains, previously shown to exhibit antimicrobial properties, i.e. the wine-isolated *L. plantarum* UFG121 [45, 51, 52], which was part of the laboratory collection, and *L. plantarum* NC8, originally isolated from grass silage, which is considered a model for several types of studies, including plantaricin production [53].

### Survival Under Oro-gastrointestinal (OGI) Stress

**Fig. 1:**
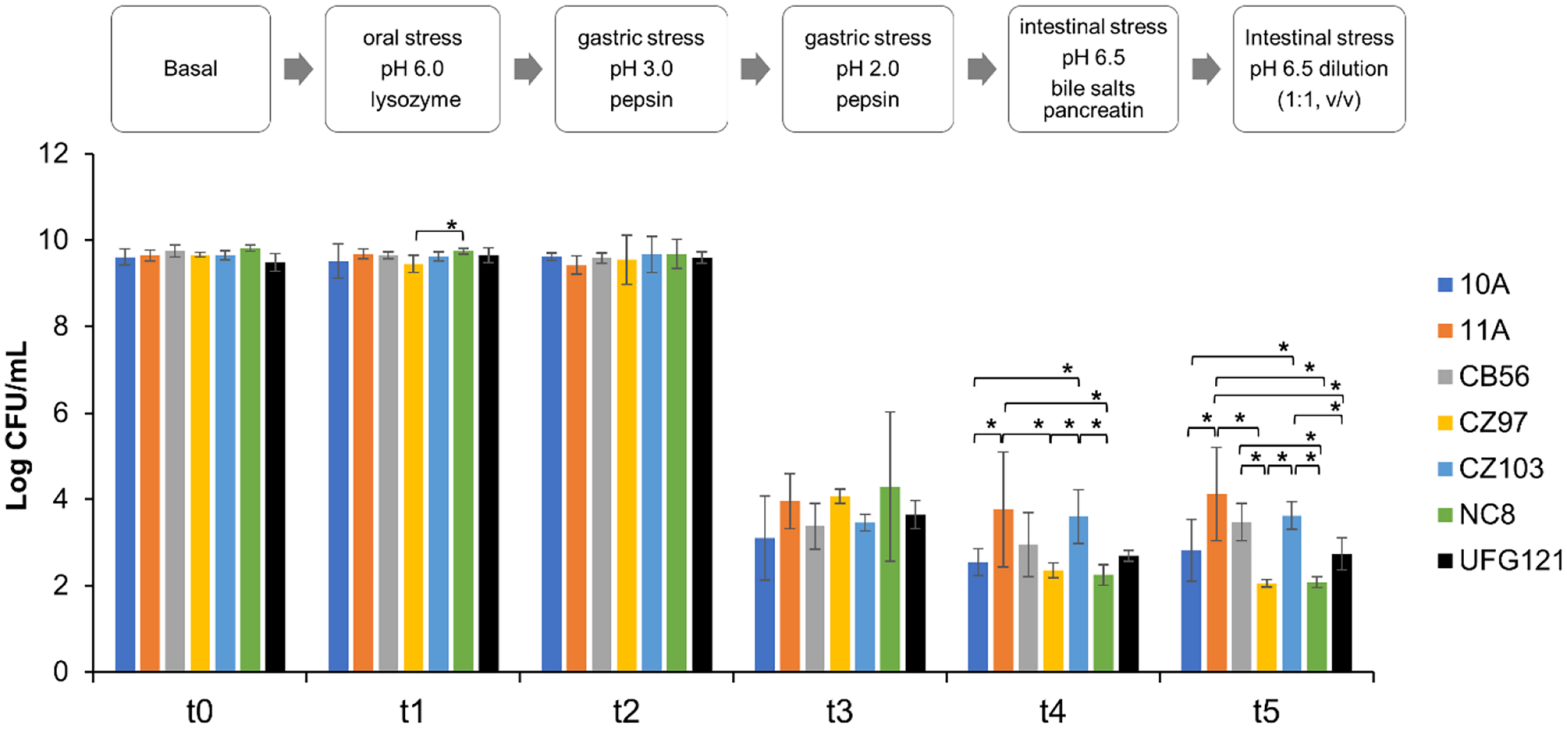
Survival of the investigated *L. plantarum* strains at different steps (in the above diagram) of the in vitro simulated OGI stress. Means ± SD of three different replicates. t1: oral stress (pH 6.0, lysozyme); t2: gastric stress (pH 3.0, pepsin); t3 gastric stress (pH 2.0, pepsin); t4: intestinal stress (pH 6.5, bile salts and pancreatin); t5: intestinal stress (pH 6.5, dilution (1:1, v/v) with an intestinal electrolyte solution). ANOVA test at each time point (*p* < 0.05), followed by Fisher’s least significant difference (LSD) test. *p < 0.05

To test their tolerance to the harsh conditions of the gut environment, the investigated strains were subjected to an in vitro model that mimics the stress found along the oro-gastrointestinal tract, and their survival is reported in Fig. 1. All strains showed quite the same good tolerance in the first two steps of the oro-gastric conditions, where the pH downshifted progressively from 6.5 to 3.0 in presence of lysozyme (oral compartment, t1) and then pepsin (gastric compartment, t2), overall retaining high viability at these stages.

Upon more acidic condition (t3), the cell viability of all strains dropped by approximately 6 logs without any significant difference among the strains; conversely, during the intestinal stress (t4, neutral pH and activity of pancreatin and bile salts), the survival rate revealed statistically significant differences between the strains, indicating the best resistance for 11A, CZ103 and CB56. During the final large intestine-mimicking phase, at neutral pH (t5), almost all *L. plantarum* strains, except CZ97, recovered cell viability compared to previous stress conditions, even better than control NC8 [53], reaching a sufficient dose to allow intestine colonisation.

### Adhesion Abilities and Biofilm Production

**Fig. 2:**
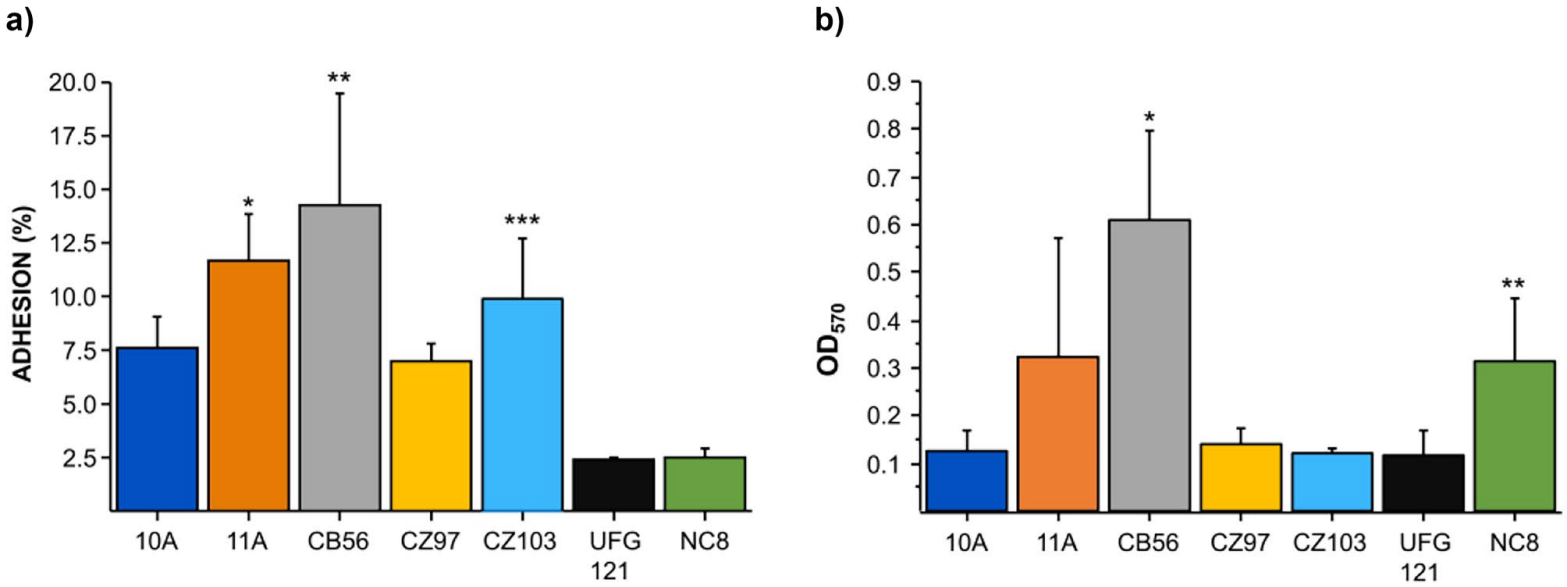
Adhesive properties of *L. plantarum* strains. **a** Adhesion to Caco-2 cells. The adhesion ability was expressed as the percentage of adhesion. *p < 0.05 vs NC8, UFG121; **p < 0.05 vs 10A, CZ97, CZ103, UFG121, NC8; ***p < 0.05 vs UFG121, NC8. **b** Ability to adhere and form biofilms on plastic surfaces. The tested *L. plantarum* strains were cultivated in 96-well plates for five days at 30 °C. To quantify the plastic-adhering biofilm, the optical density was measured (OD 570 nm). ANOVA test (*p* < 0.05) followed by Fisher’s least significant difference (LSD) test was performed for both adhesion and biofilm tests. Values represent mean ± SD of 3 different experiments. *p < 0.05 vs 10A, 11A, CZ97, CZ103, UFG121 and NC8; **p < 0.05 vs CZ103 and UFG121

In order to evaluate their potential to colonise the gut mucosa, we studied the adhesive capacities of the investigated *L. plantarum* strains, therefore testing their ability to adhere on Caco-2 cell monolayers and to form biofilms on a plastic surface (Fig. 2). Adhesion on Caco-2 cell monolayers differed significantly between the strains, with CB56 showing the highest percentage of adhesion (14.2 ± 5.2%) compared to the other strains, including the probiotic control NC8 (2.4 ± 0.4%) (Fig. 2a). Even *L. plantarum* 11A and CZ103 exhibited a good adherence to Caco-2 (adhesion rates of 11.6 ± 2.1% and 9.8 ± 2.8%, respectively), especially compared to UFG121 (2.3 ± 0.8%) and NC8, which indeed showed the lowest percentage of adhesion. All the analysed strains were able to adhere to polypropylene, showing a different ability to produce biofilms on such an abiotic surface (Fig. 2b). Statistically significant differences were observed between the investigated strains, with CB56 resulting as the best biofilm producer (OD_570_ = 0.61 ± 0.18).

### Antibiotic Resistance

In order to determine antibiotic resistance patterns and MIC, all the investigated strains were assayed for resistance towards a panel of eight antibiotics of different classes and mechanisms of action (Table 1). The strains were categorised as susceptible (S), when their growth was inhibited at a concentration of antibiotic equal to or lower than the cut-off value established by EFSA [48], or resistant (R), when capable of growing at a concentration higher than the cut-off value. All tested *L. plantarum* strains were resistant to vancomycin, streptomycin, kanamycin and gentamycin, whereas all of them were susceptible to clindamycin, tetracycline and ampicillin. *L. plantarum* 10A and CZ103 also showed susceptibility to erythromycin, whereas *L. plantarum* 11A, CZ97, UFG121 and NC8 were sensitive to chloramphenicol (Table 1).

**Table 1:** Antibiotic susceptibility. MIC (minimum inhibitory concentration) as 100% inhibition. Resistance (R) and susceptibility (S) of the tested strains was determined following EFSA guidelines [48]. Results are reported as mean ± SD of three replicates

Antibiotic (cutoff)	(MIC μg mL^−1^) mean ± SD						
	10A	11A	CB56	CZ97	CZ103	UFG121	NC8
Vancomycin (n.r.)^a^	R	R	R	R	R	R	R
Clindamycin (4)	S (0.75 ± 0.0)	S (0.55 ± 0.1)	S (0.8 ± 0.2)	S (0.32 ± 0.0)	S (0.64 ± 0.0)	S (3.75 ± 1.7)	S (1.5)
Gentamycin (16)	R (112 ± 22.6)	R (48.0 ± 0.0)	R (30.0 ± 2.8)	R (48.0 ± 0.0)	R (48.0 ± 0.0)	R	R (192)
Erythromycin (1)	S (0.9 ± 0.0)	R (1.0 ± 0.0)	R (1.25 ± 0.3)	R (1.0 ± 0.0)	S (0.44 ± 0.0)	R (1.5 ± 0.0)	R (1.5)
Tetracycline (32)	S (3.0 ± 0.7)	S (5.0 ± 1.4)	S (3.5 ± 0.7)	S (3.5 ± 0.0)	S (3.2 ± 0.3)	S (7.0 ± 1.4)	S (5.0)
Streptomycin (n.r.)	R	R	R	R	R	R	R
Kanamycin (64)	R	R (128.0 ± 0.0)	R (128 ± 0.0)	R (192 ± 0.0)	R (88.0 ± 11.3)	R	R
Chloramphenicol (8)	R (8.0 ± 0.0)	S (2.5 ± 0.7)	R (8.0 ± 0.0)	S (4.0 ± 0.0)	R (8.0 ± 0.0)	S (7.0 ± 1.4)	S (5.0)
Ampicillin (2)	S (0.18 ± 0.0)	S (0.25 ± 0.0)	S (0.41 ± 0.0)	S (0.5 ± 0.0)	S (0.44 ± 0.0)	S (0.31 ± 0.0)	S (0.25)

^a^Antibiotics cutoff values (μg mL^−1^) to determine resistance (R) related to *L. plantarum*

### Immunomodulatory Effect of *L. plantarum* CFS

The immunomodulating activity of CFS from the investigated strains was assessed in vitro on human macrophages. Preliminary cytotoxicity tests were performed to determine the safe amount of CSF to treat macrophages (supplementary Table S2). In agreement with previous findings [3], CSF concentrations between 10 and 5% (v/v) allowed high cell viability, therefore, CFS were tested at 10% (v/v) for their capacity to modulate the mRNA and protein levels of TNF-α, IL-8, IL-12 and IL-10, i.e. cytokines involved in the inflammatory process and with immune-regulatory function [54, 55].

**Fig. 3:**
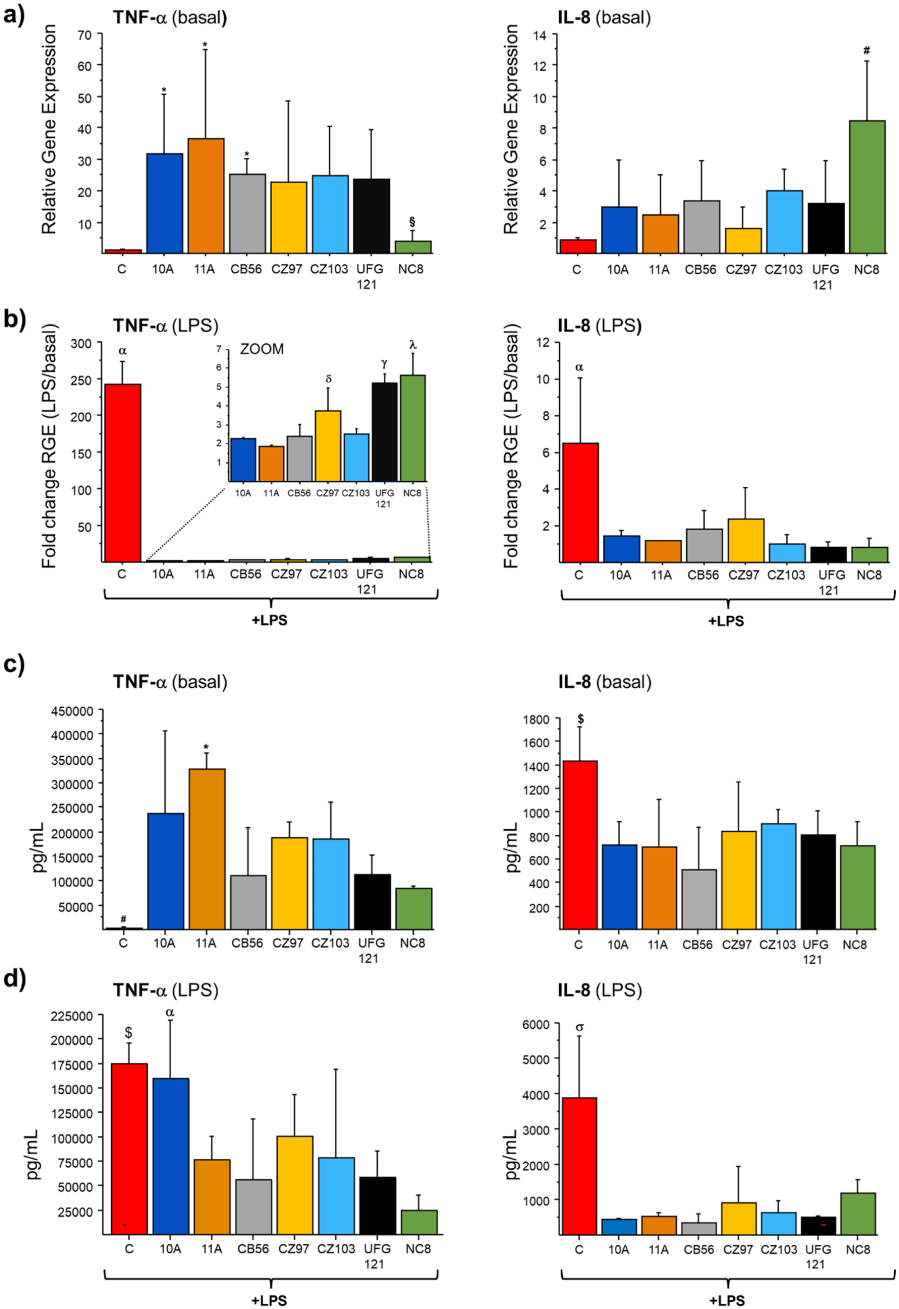
The CFS from the tested *L. plantarum* strains modulate TNF-α and IL-8 expression in macrophages under basal conditions and upon LPS stimulation. **a** Relative gene expression (RGE) of TNF-α and IL-8 genes in macrophages incubated for 24 h with the CFS from each of the indicated *L. plantarum* strains and in untreated macrophages (C), whose transcriptional level was set at 1 (basal). **b** Fold change induction of TNF-α and IL-8 transcription upon LPS-stimulation of macrophages, without (C) or with pre-incubation with CFS from *L. plantarum* strains. Fold change RGE was obtained by normalising the transcript level of LPS-stimulated macrophages to that of the corresponding non-LPS-stimulated macrophages. **c** ELISA test was used to evaluate the level (pg mL^−1^) of TNF-α and IL-8 secreted by untreated macrophages (C) and by macrophages incubated for 24 h with the CFS from each of the indicated *L. plantarum* strains (basal) and **d** by LPS-stimulated macrophages without (C) or with pre-incubation with *L. plantarum* CFS. Mean ± SD of at least two different experiments. ANOVA test (*p* < 0.05) followed by Fisher’s least significant difference (LSD) test. **a** *p < 0.05 vs C; ^§^*p* < 0.05 vs 11A; ^#^*p* < 0.05 vs C, 10A, 11A, CB56, CZ97; **b** ^α^*p* < 0.05 vs all strains; ^δ^*p* < 0.05 vs 11A; ^γ^*p* < 0.05 vs 10A, 11A, CB56, CZ97, CZ103; ^λ^*p* < 0.05 vs 10A, 11A, CB56, CZ97, CZ103. **c** ^#^*p* < 0.05 vs 10A, CZ97, CZ103 *p < 0.05 vs C, CB56, UFG121, NC8; ^$^*p* < 0.05 vs 10A, 11A, CB56, NC8; **d** ^$^*p* < 0.05 vs 11A, CB56, CZ103, UFG121, NC8; ^α^*p* < 0.05 vs CB56, NC8; ^σ^*p* < 0.05 vs all strains

The effect of CSF treatment was investigated both on the basal expression of the pro-inflammatory cytokines IL-8 and TNF-α, as well as on their induction following stimulation with LPS. Quantitative RT-PCR was used to evaluate the transcriptional levels of cytokine genes in both LPS-stimulated and non-stimulated THP-1-derived macrophages (basal conditions). As shown in Fig. 3a, the 24-h treatment with CFS, particularly those from 10A, 11A and CB56 strains, induced the pro-inflammatory cytokine TNF-α, increasing its basal expression level (from 25- to 35-fold) as compared to untreated cells (C). Upon LPS addition, i.e. a pro-inflammatory stimulus, TNF-α transcription was substantially induced in control macrophages (i.e. > 200-fold induction), whereas its fold change was much lower in cells pre-incubated with CFS from each of the strains (Fig. 3b). Indeed, in LPS-stimulated and CSF pre-treated macrophages, the transcriptional induction of TNF-α gene was from 50- to 120-fold lower, compared to LPS-stimulated macrophages without CSF pre-incubation. Although without statistical significance, the same effect was also observed for IL-8 transcription, whose induction was from 2- to 6- fold lower in CSF-pre-treated macrophages relatively to LPS-stimulated control, despite the pro-inflammatory stimulation (Fig. 3b). The expression of TNF-α and IL-8 was also studied at the protein level and, as shown in Fig. 3c, d, the concentrations of secreted cytokines were consistent with the transcriptional data, confirming the immunostimulating effects for the CFS from the investigated *L. plantarum* strains. While in THP-1 derived macrophages stimulated with LPS only, there was a dramatic increase of TNF-α (i.e. about tenfold higher after LPS addition), the secretion level was lower for LPS-stimulated macrophages preincubated with CFS, in contrast with the high basal level, detected without LPS addition. Based on TNF-α concentration, the CFS from all potential probiotics seemed to stimulate its secretion. Nevertheless, when macrophages were challenged with LPS, TNF-α production was reduced following pretreatment with CFS of strains 11A, CB56, CZ103, UFG121 and NC8. No modulation of TNF-α production was detected when macrophages were pretreated with CFS of 10A. Conversely, in relation to IL-8, all CFS could significantly attenuate the release of this pro-inflammatory cytokine, both with and without LPS stimulation.

In order to better define the immune-modulatory properties of the tested strains, we also evaluated whether CFS would modulate the expression of IL-10 and IL-12, i.e. two immune mediators with regulatory, anti-inflammatory and pro-inflammatory action, respectively. Indeed, high IL-10/IL-12 ratios, as observed in vitro, were previously demonstrated to correlate with a significant anti-inflammatory capacity of LAB in vivo [56, 57].

**Fig. 4:**
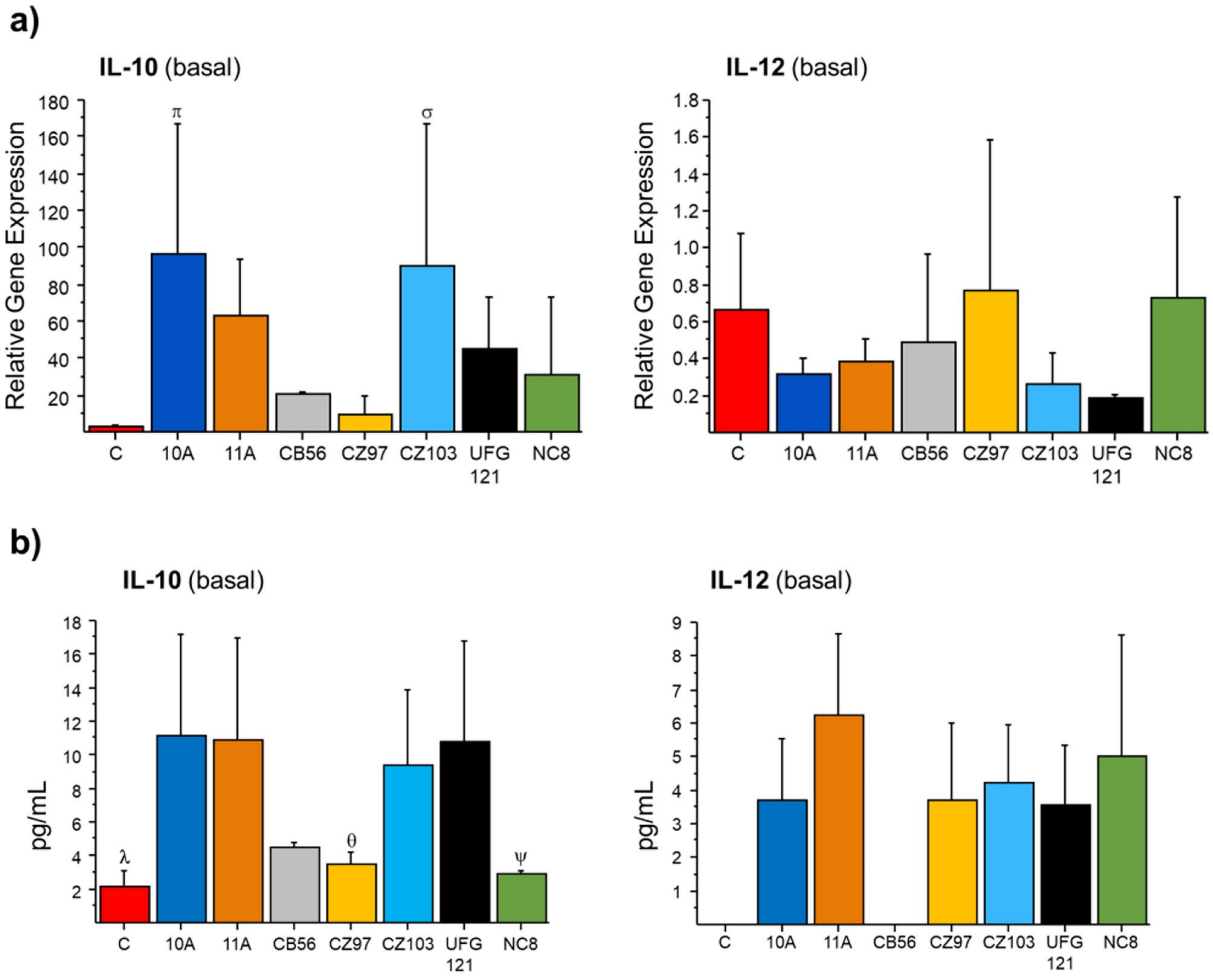
Effect of the CFS from the investigated *L. plantarum* strains on IL-10 and IL-12 gene expression and secretion by macrophages. **a** The relative gene expression (RGE) was determined by qRT-PCR, by normalising the transcriptional level of CSF-treated macrophages to that of untreated macrophages (C), whose gene expression was set at 1. **b** ELISA test was used to evaluate the level (pg mL^−1^) of secreted IL-10 and IL-12 by macrophages without (C) or with pre-incubation with CFS from each of the *L. plantarum* strains. Mean ± SD of at least two different experiments. ANOVA test (*p* < 0.05) followed by Fisher’s least significant difference (LSD) test. **a** ^π^*p* < 0.05 vs C, CB56, CZ97; ^σ^*p* < 0.05 vs C, CZ97. **b** ^λ^*p* < 0.05 vs 10A, 11A, CZ103, UFG121; ^θ^*p* < 0.05 vs 10A, 11A, UFG121; ^ψ^*p* < 0.05 vs 10A, 11A

All CFS increased IL-10 transcription, most abundantly those from 10A, 11A and CZ103 strains (Fig. 4a). Conversely, IL-12 mRNA level appeared not to be significantly affected by any CFS treatments as compared to untreated control macrophages. Consequently, the IL-10/IL-12 transcript ratio increased significantly to 270.2 ± 204.8, 211.2 ± 135.6, 312.2 ± 87.7 and 235.7 ± 129.2 (*p* < 0.05, Table 2) in the macrophages treated with CFS from strains 10A, 11A, CZ103 and UFG121, respectively, compared with untreated control macrophages (9.8 ± 13.4), indicating a potential anti-inflammatory profile for these strains. The transcriptional pattern of IL-10 and IL-12 genes mirrored the levels of the corresponding secreted cytokines, as detected in the supernatants of CFS-treated macrophages (Fig. 4b). In CFS-untreated macrophages and in macrophages treated with CFS from CB56, IL-12 levels were below the detection limit of the immunoassay, therefore, the IL-10/IL-12 protein ratio could not be calculated for these samples. Higher values of IL-10/IL-12 protein ratios were found upon treatment with CFS from *L. plantarum* 10A (4.9 ± 2.1) and UFG121 (4.3 ± 1.5), which were in good agreement with the mRNA level ratios (Table 2).

**Table 2:** IL-10/IL-12 ratio in untreated macrophages (C) and in macrophages treated for 24 h with the CFS from the *L. plantarum* strains. Values are mean ± SD of at least two different experiments. ANOVA test (*p* < 0.05) followed by Fisher’s least significant difference (LSD) test

Strains	IL-10/IL12		*P* value
	mRNA level (RT-PCR)	Secreted protein level (ELISA, pg mL^−1^)	
C	9.8 ± 13.4	n.d	
10A	270.2 ± 204.8*	4.9 ± 2.1**	*: 0.006 vs C; 0.02 vs CZ97; 0.02 vs NC8 **: 0.03 vs CZ97; 0.02 vs NC8
11A	211.2 ± 135.6*	2.5 ± 0.5	*: 0.02 vs C
CB56	23.9 ± 12.2	n.d	n.a
CZ97	42.5 ± 61.7	1.1 ± 0.9	n.s
CZ103	312.2 ± 87.7*	3.4 ± 1.6	*: 0.002 vs C; 0.02 vs CB56; 0.008 vs NC8
UFG121	235.7 ± 129.2*	4.3 ± 1.5**	*: 0.01 vs C; 0.04 vs CZ97 and NC8 **: 0.04 vs NC8
NC8	29.1 ± 35.8	0.8 ± 0.6	

## Discussion

The antibacterial properties are key determinants of probiotic action [34]; therefore, we chose this criterion to screen a group of LAB isolated from plant-related niches. Five strains, identified as *L. plantarum*, were selected as they were found to exhibit higher antagonistic activity against well-known pathogens, confirming the common good antimicrobial spectrum often observed for probiotics from this species and highlighting the strain-dependence of this feature [47, 58].

A desirable feature of probiotics is their tolerance to the harsh conditions of the gut environment [59]. By challenging the investigated strains through an in vitro model of the OGI tract, we observed a good tolerance to oral stress, which agrees with previous studies on *L. plantarum* species [3, 60]. Our data also confirm that the gastric sector, with its high acidity, represents a major barrier for orally ingested lactobacilli [61]. Moreover, the recovery of cell viability under intestinal conditions seems in accordance with previous works that compared the survival of different probiotics upon simulated OGI stress [62, 63], with *L. plantarum* exhibiting greater resistance to acids and bile, relative to other LAB species [3, 64].

Binding the intestinal mucosa is another criterion for selecting probiotics, as it indicates their potential to initiate colonisation and persist in the gut, thereby reinforcing the intestinal barrier. Human enterocyte-like Caco-2 cells are a well-accepted in vitro model for evaluating such property [62, 65, 66]. Therefore, all tested strains were assayed for adhesion on Caco-2 monolayers. The adhesion capacity of CB56 and 11A strains resulted higher than other *L. plantarum* probiotic candidates previously tested [3, 47, 66] and in the range of values observed for well-known probiotics [49, 63, 67, 68]. The biofilm-forming capacity of LAB confers protection against hostile environmental conditions and has the potential to delay pathogens’ growth [69–71], e.g. preventing biofilm-producing uro-pathogens [72]. In this study, all strains were investigated for their ability to adhere and form biofilms on plastic surfaces, which can be considered a surrogate marker of the capacity to colonise the gut for long term [73, 74]. Significant differences between biofilm levels were observed, confirming that this feature is strain-specific [75]. Overall, the data obtained from OGI transit, adhesion and biofilm assays indicate that all the tested *L. plantarum* strains, mainly 11A, CB56 and CZ103, could survive passage through the human gastrointestinal tract, adhere to intestinal cells and possibly form biofilm to begin gut colonisation.

The antibiotic susceptibility is a safety aspect that has to be considered in the evaluation of candidate probiotics. Overall, the antibiotic resistance pattern, observed here at the phenotypic level, mirrors that known for other lactobacilli [76, 77]. Like other LAB, *L. plantarum* meets the criteria established by EFSA for Qualified Presumption of Safety (QPS) [28]; moreover, it is generally recognised as safe (GRAS) by the US FDA (U.S. Food and Drug Administration. GRAS Notices. 2022) [78]. Nevertheless, some concerns have been raised recently about the safety of probiotics for human dietary consumption, as they might be a dangerous reservoir of transferrable antibiotic-resistance genes [77, 79]. In this regard, it is worth noting that the phenotypic analysis reported in this work is only indicative and shall be complemented by genotypic studies assessing both the presence and the genomic localisation of antibiotic resistance genes (i.e. an association with mobile genetic elements, indicating more concrete risks for horizontal gene transfer, with the consequent spreading of resistance) [76, 80–83].

Probiotic bacteria, including *L. plantarum*, produce bioactive substances that are released into the culture medium during growth [34]. Accordingly, *L. plantarum* CFS have been shown to have antimicrobial, anti-inflammatory [84, 85], immunomodulating [86], antioxidant [87], antitumor [88, 89] and wound-healing [85, 90, 91] properties. It is still unclear whether CFS, though containing functional probiotic-derived metabolites, can be called postbiotics, since the definition of the latter is not fully shared within the scientific community [4, 5]. Nevertheless, postbiotics are gaining a great deal of attention for their numerous advantages compared to probiotics, including greater stability, easier handling and storage, both in food and in pharmaceutical manufactures, also considering their enhanced suitability for immunocompromised and allergic patients [92, 93]. In light of this, we assessed the immunomodulating activity of CFS from the investigated strains. In earlier studies, the CFS obtained from several species of lactobacilli, including *L. plantarum*, were proved to have immune-stimulatory activity and to modulate cytokines expression in human [3, 7, 94, 95] and murine [96] immune cells, thus representing potential adjuvants in anti-inflammatory therapies. Interestingly, *Lactobacillus rhamnosus* CFS was found to be more effective than the live probiotic cells in suppressing the secretion of pro-inflammatory cytokines by dendritic cells [94], thus pointing to the use of postbiotics as an effective and safer alternative to live bacteria.

In our experimental model, the gene encoding the pro-inflammatory mediator TNF-α was significantly up-regulated by the CFS of some of the investigated *L. plantarum* strains. However, and interestingly, the same CFS would also attenuate, at the transcriptional level, the pro-inflammatory stimulation by LPS. In the same way, all CFS down-regulated IL-8 expression in macrophages challenged by LPS. At the same time, when looking at the level of secreted cytokines, a prolonged exposure of macrophages to CFS resulted in a significantly augmented release of TNF-α and a decrease of IL-8. A TNF-α increase was already observed for some lactobacilli, including *L. plantarum,* though to a different extent, probably reflecting the various conditions used (i.e. viable or inactivated cells instead of CFS, different concentration of probiotics, diverse types of immune cells, incubation times, etc.) [95, 97, 98]. It was found that the CFS from LAB, such as *L. plantarum*, induce cytokine production in THP-1 macrophages, influence the polarisation of macrophages and activate Toll-like receptor 2 (TLR2) signaling in a species- and strain-dependent manner [7]. More recently, it was demonstrated that LAB CFS stimulate the phagocytosis of murine macrophages and enhance the expression of immunomodulators, such as TNF-α, by activating the NF-ĸB and MAPK pathways [96].

The immunomodulating effect we observed on LPS-stimulated macrophages could be ascribed to some *L. plantarum* CFS components, possibly competing for the activation of the same inflammatory pathways, albeit reacting with cell receptors different from those recognising LPS [99–101]. Indeed, the engagement of the same classes of innate pattern recognition receptors, such as Toll-like receptors (TLRs), which can bind both Gram-negative and lactobacilli-derived molecules [7, 102], is thought to train the immune system towards a differentiated reaction against either commensal or potentially pathogenic microbes, thus contributing to gut immune homeostasis [20, 42, 101]. Neither the chemical nature of the immunomodulating fraction of CFS, nor the pathways they elicit on host cells, have been investigated in the present work. However, some previous studies have explored both these aspects. For instance, it was suggested that the bioactive components of *L. plantarum* CFS, endowed with TLR-engaging properties, might be proteinaceous, heat stable compounds [7]. In earlier reports, a soluble protein secreted by *Lactobacillus rhamnosus* GG was found to regulate intestinal epithelial cell growth by activating the epidermal growth factor receptor (EGFR) [103]. Conversely, the CFS from another *L. rhamnosus* strain was found to contain bioactive molecules of low molecular weight, possibly LTA, that modulated chemokine production through interactions with intestinal epithelial cells [104].

Although there are a few earlier studies specifically looking at the effects of *L. plantarum* CFS on THP-1-derived macrophages [7], an inhibited cytokine expression was seen in murine LPS-stimulated macrophages [105, 106] and other cell types, following incubation with CFS or metabolites from *L. plantarum* strains [8, 106]. Most researches have shown the anti-inflammatory effect of *L. plantarum* as whole cells, pointing to a down-regulated transcription of TNF-α and/or IL-8 in LPS-stimulated macrophages [60, 107, 108], which corresponded to decreased levels of secreted proteins [109]. In our study, only IL-8 secreted levels were significantly reduced using CFS from all strains. A differential induction, depending both on the specific proinflammatory cytokine and on postbiotic concentration, was recently described using lysates from a probiotic *L. plantarum* strain [110], pointing to a high complexity of the immunoregulative patterns.

Among the cytokines analysed in this study, IL-10 is known to suppress IL-12 production, downregulate antigen presentation and inhibit macrophages’ activation, therefore reducing the production of pro-inflammatory mediators [111]. Noteworthy, the ratio between these two cytokines (i.e. IL-10/IL-12) may be an important predictive index of the immune modulatory capacity of bacteria; hence, it can be used for screening probiotics [112], allowing the identification of strains with distinct pro-inflammatory and anti-inflammatory profile [20]. Indeed, a high IL-10/IL-12 score was previously associated with the ability of LAB strains to suppress immune responses in cell models, even in absence of pro-inflammatory stimulus [56, 112, 113], as well as in colitis models, in vivo [56, 114]. Overall, we found concentrations of secreted IL-10 and IL-12 that were lower than those reported by some previous analogous studies [56, 97], though values may be quite variable depending on lactobacilli species and treatment type [95]. Yet, we observed a good correspondence between transcript and secreted levels. Treatment with CFS from strains 10A and UFG121 resulted in significantly higher IL-10/IL-12 ratios, and, assuming these are still preliminary data, an anti-inflammatory potential for these two strains could be better defined by future studies, both in vitro and in animal models.

LAB secrete various types of soluble molecules, including peptides, proteins and short-chain fatty acids, for which an immunomodulatory activity has been previously demonstrated [9, 10]. CFS from different LAB were found to significantly induce IL-10 secretion by PBMC-derived macrophages before or after LPS challenge [95], providing evidence for the anti-inflammatory potential of LAB postbiotics. However, a few investigations reported the modulation of IL-10 and/or IL-12 specifically provided by *L. plantarum* CFS on human macrophages, finding anti-inflammatory [7] or pro-inflammatory effects [115]. As well, co-cultures of THP-1 cells with *L. plantarum* strains from different sources showed a different modulation of IL-10 expression [60, 109, 113], which points to the strain-specificity and multifactorial influence of the immune-phenotype, as also observed in our experiments [116].

The ability of some of the tested CFS to elicit anti-inflammatory signals, though weak in terms of IL-10 concentration, may be important for future applications. Indeed, these types of cytokines are known to play a role in chronic gastrointestinal disorders, and their modulation by probiotics has been observed in patients with ulcerative colitis and in animal models of colitis [56, 117, 118]. At the same time, CFS themselves were found to elicit some pro-inflammatory signals, such as TNF-α, but to reduce IL-8 production, while attenuating their LPS-induced transcription. This depicts quite an intricate scenario, where the action of the tested microbial-derived supernatants is still hard to be deciphered. In fact, because of the lack of in vivo experiments, the data gathered in the present work do not allow us to draw more definite conclusions about the immune regulating action of the tested CFS.

In summary, we show here that plant-derived *L. plantarum* strains comply with some relevant criteria of candidate probiotics and that their CFS exhibit immune-modulatory effects in vitro. This study presents some novel elements, such as the characterisation of candidate probiotics isolated from relatively atypical niches and their polyphasic exploration, including the effect of CFS on cytokine profile at both transcriptional and secretion levels, at basal and upon pro-inflammatory conditions. However, for a comprehensive understanding of the immune-modulatory properties of the investigated CFS, it will be useful to identify the bioactive molecules and their target receptors on host cells. Moreover, given the complexity of the in vivo context, any prospective application will need further studies in animal models.

### Supplementary Information

Below is the link to the electronic supplementary material.

Supplementary file1 Table S1 and Table S2 are supporting information that can be downloaded at (DOCX 20 KB)

## Data Availability

The data that support this study are available from the corresponding author, upon reasonable request.
