# Supplementary material for: Immunomodulatory Activity on Human Macrophages by Cell-Free Supernatants to Explore the Probiotic and Postbiotic Potential of Lactiplantibacillus plantarum Strains of Plant Origin
Source: Probiotics Antimicrob Proteins. 2023 May 18;16(3):911–26. doi: 10.1007/s12602-023-10084-4 (PMC11126452; doi:10.1007/s12602-023-10084-4)
Supplement: Supplementary file 1 — Supplementary file1 Table S1 and Table S2 are supporting information that can be downloaded at (DOCX 20 KB) [file 12602_2023_10084_MOESM1_ESM.docx]

**Supplementary Material of**

Immunomodulatory activity on human macrophages by cell-free supernatants to explore the probiotic and postbiotic potential of *Lactiplantibacillus plantarum* strains of plant origin

Maria Teresa Rocchetti^1^, Pasquale Russo^2^, Nicola De Simone^3^, Vittorio Capozzi^4^, Giuseppe Spano^3^, and Daniela Fiocco ^1,^*

**Table S1.** Antibacterial activity of the isolated LAB strains against food-borne pathogenic bacteria. Values are expressed as the radius (mm) of the inhibition halos. (+) Inhibition zones between 1-3 mm; (++) inhibition zones between 3-5 mm; (+++) inhibition zones greater than 5 mm. Accession codes of 16s rDNA sequences deposited in GenBank were reported only for the 5 selected strains, highlighted in bold.

| **Strain** | **Source** | ***Accession Number*** | ***Indicator target strain*** | | |
| --- | --- | --- | --- | --- | --- |
|  |  |  | ***L. monocytogenes* CECT 4031** | ***E. coli* UFG77** | ***S. aureus* UFG141** |
| *Weissella cibaria* NCE-G1 | medlar |  | +++ | ++ | ++ |
| *L. plantarum* PAN-01 | sourdough |  | ++ | - | - |
| ***L. plantarum* 10-A** | **aloe** | **ON584756** | **+++** | **++** | **+++** |
| ***L. plantarum* 11-A** | **aloe** | **ON584769** | **+++** | **+** | **++** |
| ***L. plantarum* CB-56** | **carob** | **ON585118** | **+++** | **++** | **++** |
| ***L. plantarum* CZ-97** | **strawberry tree fruits** | **ON585707** | **+++** | **++** | **++** |
| ***L. plantarum* CZ-103** | **strawberry tree fruits** | **ON598622** | **+++** | **++** | **++** |
| *L. plantarum* CZ-99 | strawberry tree fruits |  | ++ | + | ++ |
| *L. brevis* GPA-P3 | mulberry |  | ++ | + | + |
| *L. plantarum* Lp1 (641) | mulberry |  | - | + | - |
| *L. plantarum* Lp2 (843) | mulberry |  | + | + | + |
| *L. plantarum* Lp3 (844) | mulberry |  | + | ++ | + |
| *L. plantarum* Lp4 (848) | mulberry |  | ++ | ++ | ++ |
| *L. plantarum* Lp5 (850) | mulberry |  | ++ | ++ | ++ |
| *L. plantarum* LpM | sourdough |  | ++ | ++ | + |

**Table S2**. Cytotoxicity tests. Effects of different percentages (10% and 5% (v/v)) of bacterial cell-free culture supernatants (CFS) on the viability of THP-1 macrophages. Cells were cultured in presence of CFS from the indicated strain for 24 h. Relative cell viability (%) = [(OD_595_ sample – OD_595_ blank)/ (OD_595_ control – OD_595_ blank)] x 100. Mean and SD from at least 2 experiments performed in triplicates.

|  | **Relative cell viability** | |
| --- | --- | --- |
| ***L. plantarum* strains** | **10% CFS** | **5% CFS** |
| 10A | 87.56±15 | 92.77±17 |
| 11A | 72.4 ±22 | 91.6 ±24 |
| CB56 | 99.9 ±28 | 95.4 ±14 |
| CZ97 | 108.9±33 | 106.0 ±36 |
| CZ103 | 92.6±20 | 101.8 ±26 |
| UFG121 | 77.6 ±29 | 97.4 ±14 |
| NC8 | 104.5 ±6 | 114.3 ±26 |
